# Structure of the catalytic domain of the colistin resistance enzyme MCR-1

**DOI:** 10.1186/s12915-016-0303-0

**Published:** 2016-09-21

**Authors:** Vlatko Stojanoski, Banumathi Sankaran, B. V. Venkataram Prasad, Laurent Poirel, Patrice Nordmann, Timothy Palzkill

**Affiliations:** 1 Verna and Marrs McLean Department of Biochemistry and Molecular Biology, Baylor College of Medicine, Houston, TX 77030 USA; 2 Department of Pharmacology, Baylor College of Medicine, Houston, TX 77030 USA; 3 Berkeley Center for Structural Biology, Molecular Biophysics and Integrated Bioimaging, Lawrence Berkeley Laboratory, 1 Cyclotron Road, Berkeley, CA 94720 USA; 4 Department of Medicine, Medical and Molecular Microbiology “Emerging Antibiotic Resistance” Unit and European INSERM Laboratory, IAME, University of Fribourg, Fribourg, Switzerland; 5 University of Lausanne, University Hospital Center, Lausanne, Switzerland

## Abstract

**Background:**

Due to the paucity of novel antibiotics, colistin has become a last resort antibiotic for treating multidrug resistant bacteria. Colistin acts by binding the lipid A component of lipopolysaccharides and subsequently disrupting the bacterial membrane. The recently identified plasmid-encoded MCR-1 enzyme is the first transmissible colistin resistance determinant and is a cause for concern for the spread of this resistance trait. MCR-1 is a phosphoethanolamine transferase that catalyzes the addition of phosphoethanolamine to lipid A to decrease colistin affinity.

**Results:**

The structure of the catalytic domain of MCR-1 at 1.32 Å reveals the active site is similar to that of related phosphoethanolamine transferases.

**Conclusions:**

The putative nucleophile for catalysis, threonine 285, is phosphorylated in cMCR-1 and a zinc is present at a conserved site in addition to three zincs more peripherally located in the active site. As noted for catalytic domains of other phosphoethanolamine transferases, binding sites for the lipid A and phosphatidylethanolamine substrates are not apparent in the cMCR-1 structure, suggesting that they are present in the membrane domain.

**Electronic supplementary material:**

The online version of this article (doi:10.1186/s12915-016-0303-0) contains supplementary material, which is available to authorized users.

## Background

The increasing prevalence of antibiotic resistance among bacterial Gram-negative pathogens is a serious threat to global health. A particular problem is related to the spread of multidrug resistant Gram-negative bacterial infections belonging to the enterobacterial family that are responsible for the highest number of infections for human kind. The rapid increase in carbapenem-resistant *Enterobacteriaceae* that produce carbapenemase enzymes, such as KPC and NDM [1], is of special interest. Due to the paucity of novel antibiotics, polymyxins (colistin, polymyxin B), although introduced in the 1950s, are gaining a renewed interest for treating infections due to said multidrug-resistant infections. Polymyxins are cationic polypeptides [2] that act by binding to the lipid A moiety of bacterial lipopolysaccharides (LPS), subsequently disrupting the bacterial membrane.

**Fig. 1:**
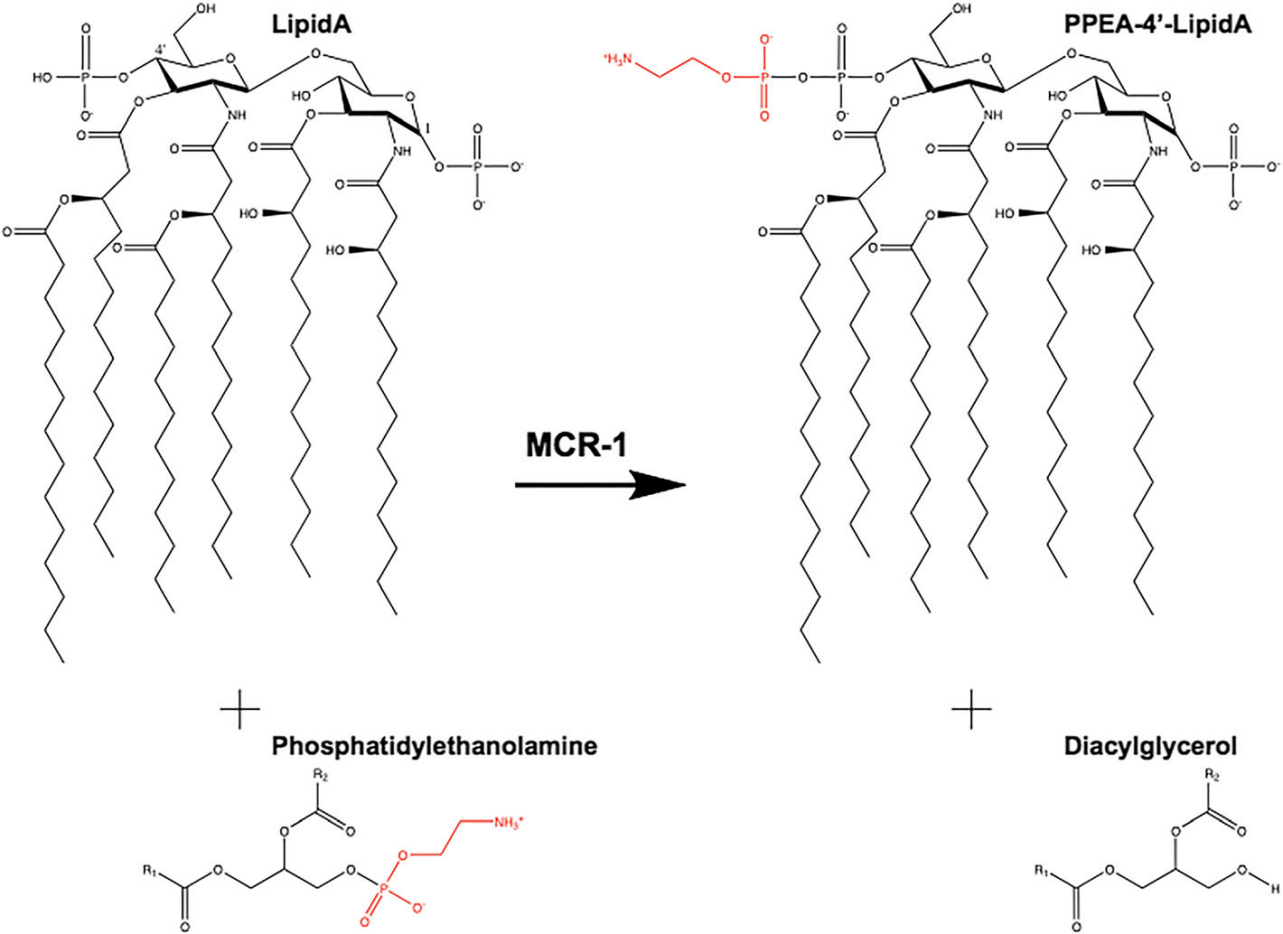
Structure of lipid A of *E. coli* showing reaction catalyzed by MCR-1. Phosphatidylethanolamine’s R1 and R2 groups are constituted of acyl chains. The phosphoethanolamine moiety that is transferred from phosphatidylethanolamine to lipid A is shown in red. In this reaction, the transfer has been shown to occur to the 4’ position of lipid A, however, transfer can also occur to the 1’ position

Acquired and chromosome-encoded resistance to colistin has been reported among Gram-negative bacteria and some species, such as *Neisseria spp.* and *Serratia spp.*, are intrinsically resistant to colistin [3]. The most common mechanism of acquired resistance involves modification of the LPS component of the outer membrane. Specifically, resistance occurs due to modification of the 1’ and 4’ phosphate groups of lipid A to neutralize the negative charge and reduce binding of the positively charged colistin [3, 4]. The phosphates are modified with 4-aminoarabinose by the aminoarabinose transferase ArnT or by the addition of phosphoethanolamine (PEA) by PEA transferase enzymes (Fig. 1) [5–7]. Chromosome-encoded and acquired resistance to polymyxins is associated with mutations found in genes for two-component regulatory systems and result in expression of the transferase enzymes that modify LPS [3, 4].

The X-ray structures of an ArnT transferase as well as the catalytic domain of the *Neisseria meningitidis* (LptA) and *Campylobacter jejuni* (EptC) PEA transferases have been determined [8–10]. ArnT is a membrane protein with a periplasmic domain and is a member of the GT-C family of glycosyltransferases, while the PEA transferases have a membrane-spanning domain and a periplasmic catalytic domain [8–10]. The catalytic domain of the LptA and EptC PEA transferases have a similar structure and are members of the sulfatase group with a fold similar to alkaline phosphatase [9, 10].

Very recently, a plasmid-encoded LPS-modifying enzyme, named MCR-1, that provides colistin resistance has been reported from *Enterobacteriaceae* in China [11]. This is an additional source of concern since it is the first transferrable resistance to polymyxin antibiotics. It raises the specter of transferable pan-drug resistance in *Enterobacteriaceae.* Indeed, there are also recent reports of the spread of the same *mcr-1* gene worldwide in community- and hospital-acquired pathogens in humans and in animals [12–16]. The MCR-1 enzyme is 41 % and 40 % identical to the PEA transferases LptA and EptC, respectively, and sequence comparisons suggest the active-site residues are conserved [11]. Here, we report the X-ray crystal structure of the soluble, periplasmic catalytic domain of the MCR-1 enzyme determined at 1.32 Å resolution. The fold of the MCR-1 catalytic domain is similar to that of the LptA and EptC transferases, as expected based on sequence homologies. In addition, many of the presumed active-site residues are conserved, although the number and position of active site zinc ions differ among the structures. The position of binding sites for lipid A and phosphatidylethanolamine are not apparent in the catalytic domain structure, suggesting that they are present in the membrane domain.

## Results

### Crystal structure of the catalytic domain of MCR-1

**Fig. 2:**
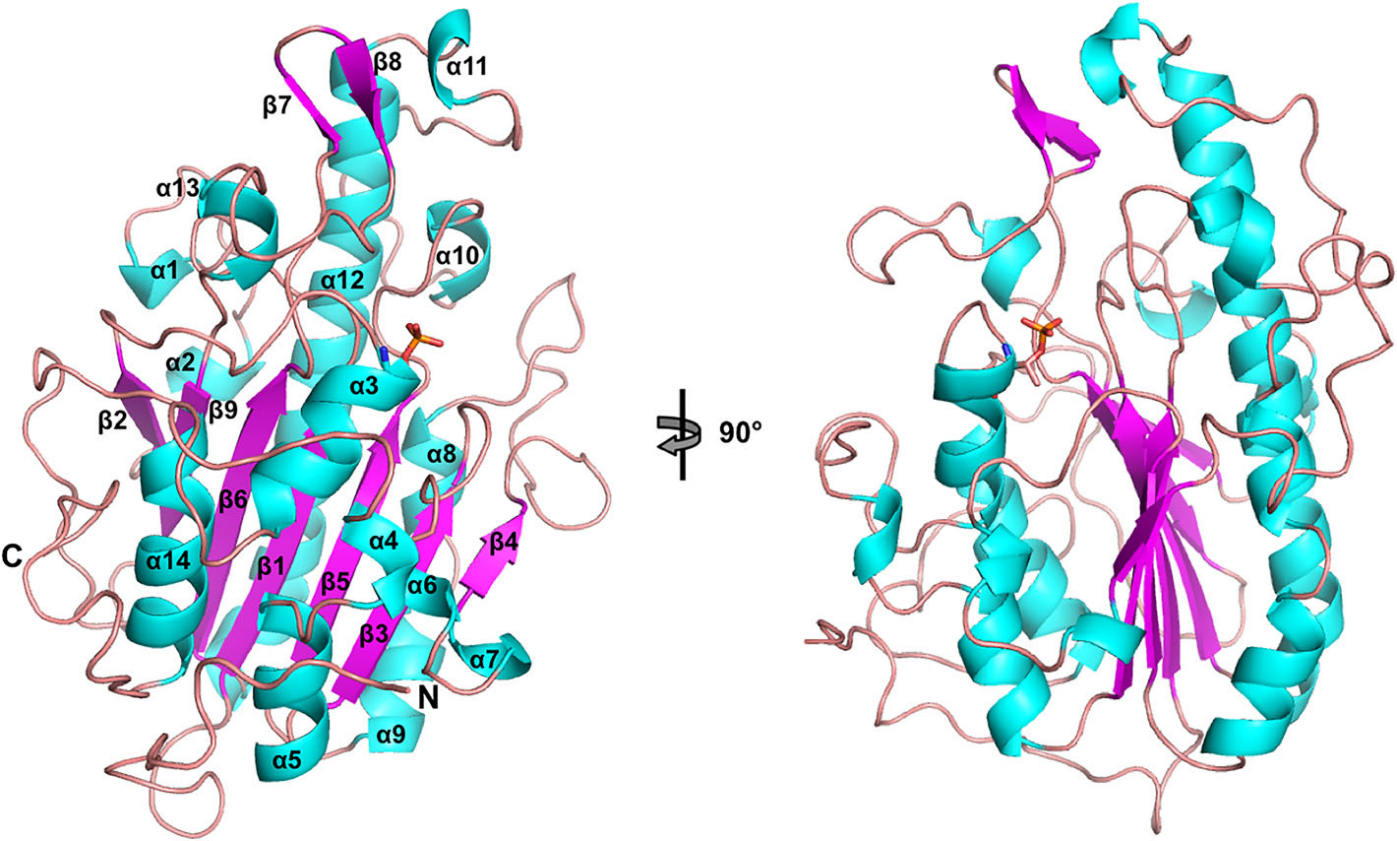
Structure of the catalytic domain of MCR-1 phosphoethanolamine transferase. Secondary structure elements are depicted as a ribbon model and colored in cyan (helices), purple (strands), and salmon (loops). Additionally, the phosphothreonine 285 is represented in sticks. Right, rotated 90 °, a view of the characteristic alkaline phosphatase α/β/α fold of cMCR-1

In order to examine the structural and molecular features of the catalytic domain of MCR-1 (cMCR-1, residues 215–541), we determined its X-ray crystal structure (Fig. 2). Two soluble domain truncations of cMCR-1, *mcr-1*_Δ1–214_, and *mcr-1*_Δ1–236_, were produced with an N-terminal His_6_-tag sequence (see Methods). It was found that the *mcr-1*_Δ1–214_ construct produced sufficient amounts of protein for purification and crystallographic studies. Diffraction-quality crystals of cMCR-1_Δ1–214_ were obtained only after cleavage of the N-terminal His_6_-tag. Excluding the His_6_-tag, the cMCR-1_Δ1–214_ protein consists of 330 residues with an estimated molecular weight of 36.8 kDa. The crystals diffracted to 1.32 Å resolution and the phases of cMCR-1 were determined by single anomalous diffraction techniques using zinc as the anomalous scatterer. The cMCR-1 protein crystallized in the P4_3_2_1_2 space group with one molecule in the asymmetric unit (Table 1). The determined structure shows a well-defined electron density for a total of 324 residues spanning from Asp218 to Arg541.

**Table 1:** Data collection, phasing, and refinement statistics for the structure of cMCR-1

	cMCR-1 (PDB ID 5K4P)
Data collection	
Space group	P4_3_2_1_2
Cell dimensions	
*a*, *b*, *c*, Å	59.1, 59.1, 186.7
*α*, *β*, *γ*, °	90, 90, 90
Wavelength, Å	1.00
Resolution, Å^a^	49.9–1.32 (1.39–1.32)
*R*_merge_	0.057 (0.491)
*R*_meas_	0.079 (0.892)
*R*_pim_	0.055 (0.552)
*I/*s(*I*)	9.6 (1.7)
*CC*_1/2_	0.996 (0.547)
Completeness, %	100 (100)
Redundancy	14.7 (11.7)
Refinement	
Resolution, Å	49.9–1.32 (1.39–1.32)
No. reflections	78753
*R*_work_/*R*_free_	0.1464/0.1732
No. atoms	2935
Protein	2541
Ligand/ion (D-sorbitol/zinc)	12/10
Water	368
*B* factors, Å^2^	
Protein	12.1
Zinc	10.8
D-sorbitol	11.3
Water	25.1
Root-mean-square deviations	
Bond lengths, Å	0.007
Bond angles, °	0.878
MolProbity clash score	1.00

^a^Values in parentheses are for highest resolution shell

cMCR-1 is a globular protein with an overall hemispherical shape and a centrally located β-sheet composed of seven β-strands sandwiched between α-helical structures (Fig. 2). The catalytic domain of MCR-1 assumes the α/β/α fold characteristic of the alkaline phosphatase superfamily. The first 23 residues of cMCR-1 constitute a random loop region that wraps around the surface of the protein. Although unstructured, this region was found to be important for the expression and purification of the cMCR-1 protein in that the removal of this region (MCR-1_Δ1–236_ construct) resulted in aggregation and poor purification yields (data not shown).

**Fig. 3:**
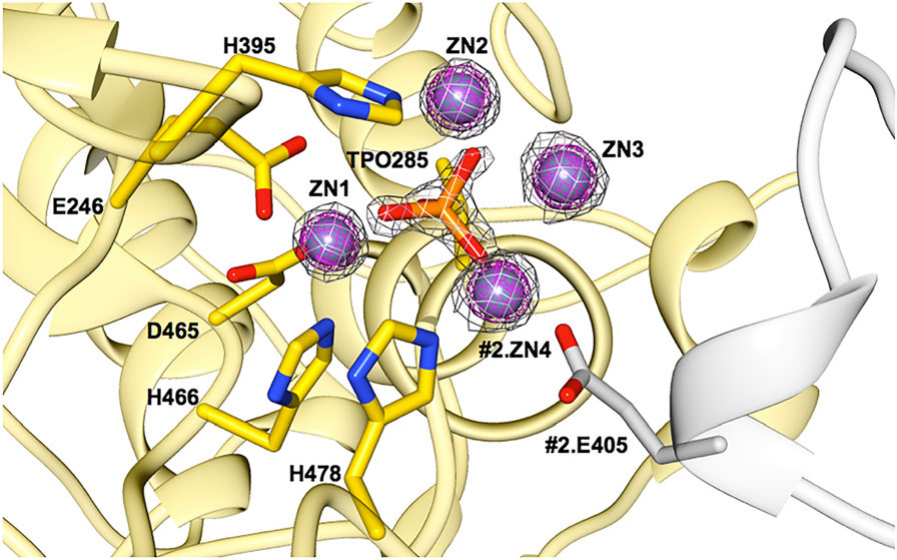
Structure of the active-site phosphothreonine with associated zinc ions. The phosphothreonine (TPO285) is represented as a yellow-orange-red stick model and the zinc ions (ZN1, ZN2, ZN3, and ZN4) that surround the phosphothreonine are shown as slate blue spheres. The 2F_o_ − F_c_ simulated annealing difference map of the final refined model contoured at σ = 4.0 is shown as a gray mesh. ZN4 is also coordinated by Glu405 from a neighboring molecule in the crystal. The neighboring MCR-1 protein is colored white and labeled with the prefix #2

**Fig. 4:**
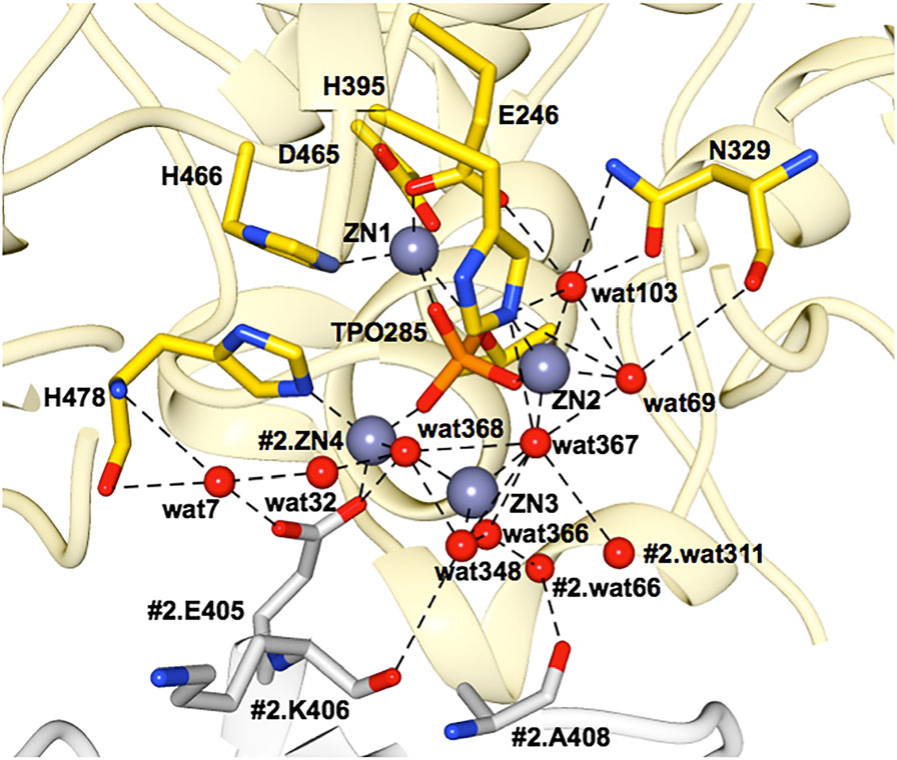
Representation of the zinc ions identified in the active site of cMCR-1. Zinc ions are shown as slate blue spheres and active-site residues are represented in stick model. In yellow, is one MCR-1 (#1) molecule, and in white, is another MCR-1 (#2) molecule located adjacent to the first one. ZN4 from the second molecule is positioned at the interface and is shared by the two molecules. Structural water molecules are labeled and hydrogen bonds and zinc interactions are shown with dashed lines

Upon inspection of the electron density map, a residual density was observed protruding from the side chain hydroxyl oxygen of Thr285. The biological function of the enzyme and the tetrahedral shape of the density suggested the presence of a phosphate group covalently attached to the side-chain oxygen resulting from phosphorylation of Thr285 (Figs. 3 and 4). The phosphate group was then modeled into this density followed by positional and occupancy refinement, and validation by using difference density maps. The average occupancy and B-factor of the phosphate group atoms following the refinement were 0.97 and 9.0 Å^2^, respectively, indicating that nearly all of the enzyme was in the phosphorylated state. The phosphothreonine 285 is located centrally on the flat surface of the hemisphere opposite to the starting β-strand β1, at the N-terminal end of the α3 helix (Fig. 2). Surrounding the phosphothreonine are three zinc ions, which were identified by the anomalous signal and further confirmed by difference maps (Figs. 3 and 4). Based on previous studies of PEA transferases, the presence of phosphorylated threonine, and the zinc binding sites, this region of the catalytic domain was inferred as the active site of MCR-1 [9, 10].

**Fig. 5:**
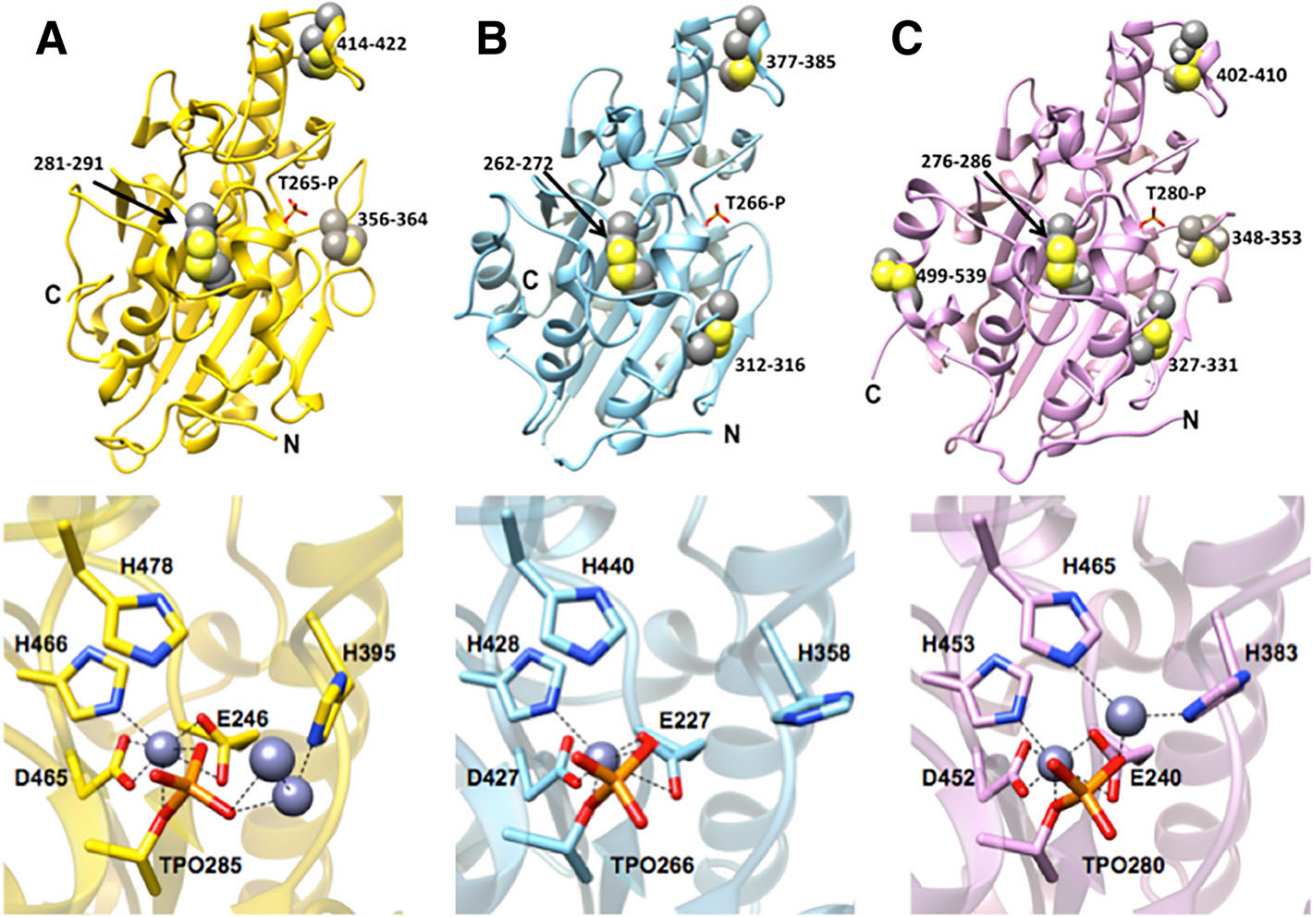
Comparison of the crystal structures of the catalytic domains of MCR-1 (**a**), EptC (*C. jejuni*) (PDB code 4TN0) (**b**), and LptA (*N. meningitidis*) (PDB code 4KAY) PEA transferases and their conserved active-site residues (**c**). Top panels – All of the enzymes adopt a similar fold and the active-site threonine is in phosphorylated form. The active site phosphothreonine is labeled for each enzyme. Disulfide bonds are shown as space-fill spheres and the numbers of the participating residues are labeled. Bottom panels – Representative active-site residues are shown in stick model together with interacting zinc ions represented as slate blue spheres. The dashed lines represent interacting distances < 3.3 Å. The MCR-1 active site has three zinc ions bound compared to one zinc for EptC and two zincs for LptA. The MCR-1 zinc ion that is coordinated by phosphothreonine-285, Glu246, Asp465, and His466 is conserved in EptC and LptA

The catalytic domain of MCR-1 contains six cysteine residues and the cysteines form three disulfide bonds between residues Cys281/Cys291, Cys356/Cys364, and Cys414/Cys422 (Fig. 5a). The location of the catalytic domain of full-length MCR-1 is presumably on the periplasmic face of the cytoplasmic membrane, based on the predicted five membrane spanning helices in the N-terminal transmembrane domain of the protein and previous results with PEA transferases. The cMCR-1_Δ1–214_ construct used for protein expression, however, lacks the transmembrane domain and is expressed in the reducing environment of the cytoplasm. It is possible that, upon cell lysis for purification, the cysteines become oxidized to form the disulfide bonds. The Cys281/Cys291 disulfide bond bridges a loop region with the α3-helix on which the phosphothreonine is located. The Cys356/Cys364 bond is located in an extended loop region that connects the β4-strand and α8-helix and the Cys414/Cys422 disulfide bond is positioned near each end of the α11-helix (Fig. 5a).

### Active-site and protein surface zinc binding sites

There are ten zinc ions in total per protein molecule in the crystal structure of cMCR-1. Three zinc ions (Zn1, 2, and 3, with average occupancies of 0.97, 0.88, and 0.93 and B-factors of 6.6, 7.1, and 8.7 Å^2^, respectively) are located in the active site of the enzyme as well as an additional zinc ion (Zn4, with average occupancy of 0.95 and B-factor of 6.8 Å^2^) that is shared between two adjacent protein molecules in the crystal lattice (Fig. 4). Zn1 is buried in the active site of MCR-1 and is tetrahedrally coordinated by a phosphate oxygen of phosphothreonine-285 as well as the conserved residues Asp465, Glu246, and His466 (Fig. 4). Zn2 is bound by a phosphate oxygen of phosphothreonine-285, Nε2 atom of His395, and three waters in a trigonal bipyramidal configuration. Zn3 is less embedded in the enzyme and is tetrahedrally coordinated by four water molecules. The waters that coordinate Zn2 and Zn3 are connected to the protein by an extensive hydrogen bond network. For example, water-103, involved in the binding of Zn2, is hydrogen bonded to the side chain oxygen of Glu246 and the side chain Nδ2 of Asn329 (Fig. 4). Water-103 is also hydrogen bonded to water-69, which also interacts with Zn2 (Fig. 2). In addition, water-367 forms a bridge between Zn2 and Zn3. Another Zn3 ligand, water-368, forms a hydrogen bond to Nε2 of His478 and is also hydrogen bonded to water-366 and water-348, each of which is a Zn3 ligand (Fig. 4). Therefore, the waters that coordinate Zn2 and Zn3 are part of a network of hydrogen bonds that link the zinc atoms to each other and the protein. Zn4 is also near the active site and interacts with phosphothreonine-285 and water molecules as well as Glu405 from a neighboring cMCR-1 molecule so that Zn4 serves as a bridge in a crystal contact (Fig. 4). The remaining zinc ions are scattered over the surface of the protein and are mainly coordinated by aspartate and glutamate residues. The large number of zinc ions associated with the MCR-1 structure is likely due to the fact that the enzyme crystallized in a condition containing 200 mM zinc acetate (see Methods).

### Test of the effect of a Thr285Ala substitution on MCR-1-mediated resistance

**Fig. 6:**
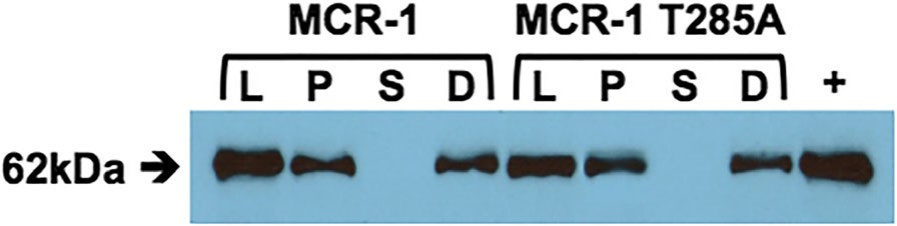
Immunoblot indicating expression levels and localization of N-terminal His-tagged, full-length wild-type MCR-1 (lane 1–4) and T285A (lane 5–8) mutant. *L* whole cell lysis, *P* pellet, *S* soluble, *D* detergent-soluble membrane fraction. Lane 9 (+) purified His-MCR-1

If Thr285 is the catalytic nucleophile, mutation of this residue would be expected to greatly decrease MCR-1 function. This was tested by mutating this residue to alanine in a plasmid encoding the full-length *mcr-1* gene with an N-terminal His-tag and measuring the minimum inhibitory concentration of colistin and polymyxin B. The strain containing wild-type *mcr-1* exhibited minimum inhibitory concentrations (MICs) of 8.0 and 6.0 μg/mL for colistin and polymyxin B, respectively, compared to a MIC of 0.032 μg/mL for the control strain containing the plasmid without *mcr-1* (Table 2). Mutation of Thr285 to alanine lowers the colistin and polymyxin B MICs to near the control levels (Table 2). Similar results were obtained with the wild-type MCR-1 gene and T285A mutant without an N-terminal His-tag, indicating the His-tag does not impair in vivo MCR-1 function. To assess whether the T285A mutant is expressed and directed to the membrane, immunoblot analysis was performed using an anti-His-tag antibody (Fig. 6). The immunoblot suggests that the T285A mutation does not affect the expression or the membrane location of the MCR-1 protein. Altogether, these results indicate that Thr285 is important for MCR-1 function and is consistent with it serving as the catalytic nucleophile.

**Table 2:** Minimum inhibitory concentrations (μg/mL) for *E. coli* containing a plasmid encoding N-terminally His-tagged wild-type MCR-1 and MCR-1 T285A

*E. coli* +	Colistin	Polymyxin B
pET28a (empty vector)	0.032	0.032
*mcr-1* pET28a	8.0	6.0
*mcr-1* T285A pET28a	0.125	0.092

### Structure homology between cMCR-1, cEptC and cLptA

The structure coordinates of MCR-1 were submitted to the DALI server for comparison with other known protein structures. The closest structural homologues are the PEA transferases EptC from *Campylobacter jejuni* and LptA from *Neisseria meningitidis* [9, 10]. These enzymes had previously been noted as homologues of MCR-1 based on primary amino acid sequence homology [11]. An amino acid sequence alignment of MCR-1 with EptC and LptA is shown in Additional file 1: Figure S1.

The cMCR-1 and cEptC enzymes are highly similar and share the majority of their secondary structures (Fig. 5a, b). The cMCR-1 and cEptC structures have a root-mean-square deviation (RMSD) of 0.861 Å between 217 residues when matching Cα atom pairs. Both cMCR-1 and cEptC contain three disulfide bonds. The MCR-1 Cys281/Cys291 disulfide bond near the catalytic Thr265 is positioned similarly to the cEptC Cys262/Cys272 bond and the cMCR-1 Cys414/Cys422 bond that is distant from the active site is equivalent to the cEptC Cys377-Cys385 pair (Fig. 5). The cMCR-1 Cys356/Cys364 pair has no equivalent in cEptC. This pair resides in an extended loop between the β4 strand and the α8 helix in cMCR-1, and this loop is significantly shorter and lacking a disulfide bond in cEptC (Fig. 5). In addition, cEptC contains a Cys312/Cys316 disulfide bond near the active site that is also conserved in the cLptA enzyme but is not present in cMCR-1 (Fig. 5). The functional consequences of these differences are currently not known.

The RMSD between 237 Cα atom pairs of the structures of cMCR-1 and cLptA is 0.875 Å. This RMSD is slightly higher than that of cMCR-1/cEptC, but nevertheless, the structures are very similar. The disulfide-bonding pattern of these two enzymes varies in that cLptA has ten cysteine residues (four more than cMCR-1) that form five disulfide bonds compared to only three in cMCR-1. The Cys281/Cys291, Cys414/Cys422, and Cys356/Cys364 disulfide bonds found in cMCR-1 all have equivalents in cLptA (Fig. 5). As noted above, the cMCR-1 Cys356/Cys364 pair is in an extended loop between the β4 and the α8 helix and this loop is truncated in cEptC. Similar to cMCR-1, an extended loop is present in this region in cLptA and contains a disulfide bond (Cys348/Cys353); however, the conformation of this loop is different than that in cMCR-1 (Fig. 5). Of the two additional disulfide pairs found in cLptA but not in cMCR-1, one is Cys327/Cys331, which is near the active site and has an equivalent in cEptC. In contrast, the second additional cLptA disulfide bond (Cys499/Cys540) has no equivalent in cEptC or cMCR-1. It is located towards the C-terminus of cLptA, where it connects two α-helices. This region is an unstructured extended loop in cMCR-1.

The active sites of MCR-1, EptC, and LptA are highly conserved (Fig. 5). All three enzymes contain a threonine residue that likely acts as a nucleophile for attack on the phosphate of the donor molecule phosphatidylethanolamine. This threonine is phosphorylated in all three structures (Fig. 5). A bound zinc (Zn1) is coordinated by the phosphothreonine in MCR-1 and this zinc is conserved in the structures of cEptC and cLptA (Fig. 5). Glu246, Asp465, and His466 also coordinate this zinc in cMCR-1 and these residues are conserved in cEptC and cLptA. Structural equivalents of this zinc and the coordinating residues are also conserved in alkaline phosphatase. Therefore, this zinc site appears to be a core component of this family of enzymes and it has been proposed to stabilize the alkoxide of the active-site threonine for nucleophilic attack on the phosphate of the phosphatidylethanolamine substrate for LptA [9].

The cMCR-1 His478 and His395 residues are also conserved in cEptC and cLptA (Fig. 5). In the cMCR-1 structure, His395 coordinates an additional zinc (Zn2) in the active site, while His478 does not contact zinc. In the cLptA structure, the His395 and His478 equivalent residues coordinate the second zinc (Fig. 5). The differences between cMCR-1 and cLptA for the His478 residue are due to the altered position of the second zinc in cMCR-1 compared to cLptA (Fig. 5). The cEptC active site contains only one zinc, so neither of these His residues coordinates zinc in the cEptC structure. Finally, as noted above, MCR-1 contains a third zinc that contacts phosphothreonine-285 but is not coordinated by other MCR-1 residues and a fourth zinc that is bridged by a neighboring MCR-1 molecule in the crystal lattice (Figs. 4 and 5). Neither of these zincs is found in the cLptA or cEptC structures [9, 10]. The peripheral location of these zincs suggests they may not play a role in catalysis and may be a function of the high zinc concentration in the crystallization conditions for MCR-1. The binding of multiple zinc ions by both cLptA and particularly cMCR-1, compared to cEptC, is likely due to increased zinc concentrations during crystallization. The functional significance of the additional zinc sites in cMCR-1 awaits further study.

## Discussion

PEA transferase enzymes, such as MCR-1, catalyze the lipid-to-lipid transfer of PEA from phosphatidylethanolamine to the 1’ or 4’ phosphate positions of lipid A [7]. The catalytic domain of MCR-1 has the alkaline phosphatase superfamily fold and there is conservation of several residues in the active site compared to alkaline phosphatase. The mechanism of MCR-1 and other PEA transferases is not known, although it may proceed similarly to alkaline phosphatase [9]. The Zn1 site in MCR-1 is common to PEA transferases and alkaline phosphatase (Fig. 5). In alkaline phosphatase, this zinc stabilizes the alkoxide form of an active site serine for nucleophilic attack on the phosphate of phosphate monoesters [17, 18]. In MCR-1, this zinc may stabilize the alkoxide form of the structurally analogous Thr285 for nucleophilic attack on the phosphate of phosphatidylethanolamine to create an intermediate with Thr285 linked to PEA. Binding of lipid A in an appropriate position for nucleophilic attack on the lipid A 1’ or 4’ phosphate on the phosphate of the Thr285-PEA intermediate could then transfer the group to lipid A. As discussed below, however, there are no obvious binding sites for phosphatidylethanolamine and lipid A on the catalytic domain and insights into the catalytic mechanism await the structure of the entire MCR-1 protein in the presence of substrates or substrate analogues.

In support of the catalytic threonine as a nucleophile for attack on a phosphate, this residue is phosphorylated in the cMCR-1, cLptA, and cEptC structures [9, 10]. This is unlikely to be a true intermediate, however, as there is no indication of electron density for ethanolamine attached to the phosphate. This may be due to expression of the protein without the membrane domain and thus it is not localized to the membrane where the lipid substrates reside.

Although the structure of cMCR-1 reveals active site similarities to alkaline phosphatase and a putative nucleophilic threonine, the binding sites for the lipid A and phosphatidylethanolamine substrates are not obvious. The MCR-1 catalytic domain has a hemispheric shape and the zinc binding pocket containing phosphothreonine-285 resides on a relatively flat surface. The structure of the ArnT aminoarabinose transferase that also uses lipid A as substrate and, like the PEA transferases, acts at the 1’ and 4’ phosphate positions of lipid A, was recently determined [8]. The aminoarabinose transferred by ArnT to the lipid A phosphates is provided by the lipid carrier undecaprenyl phosphate. Although ArnT is not homologous to the PEA transferases and the structures are not expected to be conserved, it is a lipid-to-lipid transferase with similar substrates and a similar function as PEA transferases. The ArnT apo-structure revealed cavities that could potentially bind the lipid substrates and a structure with undecaprenyl phosphate indicated a cavity that binds this substrate, thereby also suggesting the binding site of lipid A resides in another large cavity [8]. The lipid binding cavities of ArnT reside both within the membrane-spanning section and near the periplasmic interface [8]. In contrast, no such cavities are apparent in the cMCR-1 structure. An important difference between the ArnT and cMCR-1 structure experiments is that the entire ArnT protein structure was determined, including the membrane portion containing 13 membrane-spanning helices while only the periplasmic domain structure of MCR-1 was solved. It is likely that the membrane domain of MCR-1, with five predicted membrane spanning helices, contributes to the lipid binding sites, which may reside in the interface between domains as suggested previously for LptA [8, 9].

## Conclusions

The high-resolution structure of the polymyxin resistance enzyme MCR-1 has been determined at 1.32 Å resolution. The structure of the catalytic domain of MCR-1 reveals conservation of structure, particularly in the active site, with other PEA transferases [9, 10]. PEA transferases are an interesting drug target in that they are present in a wide range of Gram-negative bacteria and play a role in modifying the bacterial lipopolysaccharide in response to environmental conditions, including host defenses [7]. The structural conservation of the MCR-1 active site with other PEA transferases suggests that inhibitors of MCR-1 may also inhibit chromosomally encoded PEA transferases. Such inhibitors would not only restore polymyxin susceptibility but also modify the ability of bacteria to avoid host defenses during pathogenesis.

## Methods

### Construct design, cloning, protein expression, and MIC determination

The MCR-1 protein is predicted to consist of a membrane-spanning domain and a periplasmic catalytic domain [11]. In order to design a protein expression construct for the soluble catalytic domain, the *mcr-1* sequence was retrieved from GI: 817091896 (GeneBank: AKF16168.1). The TMHMM Server, v2.0 S, was used to identify the five potential transmembrane regions [19]. Additionally, the ThreaDom server was utilized for protein domain boundary prediction [20]. The ThreaDom results suggest that MCR-1 consists of two domains with domain 1 (N-terminal, transmembrane domain) containing residues 1–214 and domain 2 (C-terminal, soluble catalytic domain) consisting of residues 215–541. The C-terminal domain, confined by residues 215–541, was submitted to the I-TASSER server for protein structure and function prediction [21–23]. I-TASSER predicted that the first 23 residues of the soluble domain consist of an unstructured loop region with the first secondary structure (β-strand) starting at residue Arg238. Based on the aforementioned information, two different constructs were generated by PCR amplification of the 215–541 encoding region (*mcr-1*_Δ1–214_ construct) and the 237–541 encoding region (*mcr-1*_Δ1–236_ construct) of the *mcr-1* gene. The PCR products were cloned into the pET28a vector by Gibson assembly for further protein expression and purification. DNA sequencing of the entire *mcr-1* region was performed to ensure that no extraneous mutations were present.

In order to test the in vivo function of MCR-1 and the T285A mutant, the full-sized MCR-1 was cloned into the plasmid pBCKSII. In addition, the full-sized MCR-1 with an N-terminal His-tag was inserted into the pET28a plasmid. The T265A substitution was introduced into both the wild-type and His-tagged version of MCR-1 by oligonucleotide-directed mutagenesis. The DNA sequence of the entire genes was determined to ensure no extraneous mutations occurred.

Immunoblot analyses were performed with full-length MCR-1 proteins, including N-terminal His_6_-tags, and were detected with anti-His monoclonal mouse antibody conjugated to horseradish peroxidase (Qiagen, Venlo, Netherlands, Cat. No./ID 34460; Lot/Batch No. 139306078). In brief, cells were grown at 37 °C with shaking to an OD_600_ of 0.5. Protein production was induced by the addition of IPTG to a final concentration of 0.5 mM. Proteins were expressed at 25 °C with shaking and cells were harvested after 2 hours of incubation. Cells were lysed by sonication. The lysate was centrifuged to obtain the pellet and supernatant, which were used for assaying the presence of MCR-1 by immunoblotting (Fig. 6). The membrane fraction was obtained by treating the whole cell lysate with detergent (30 mM dodecyl maltoside) and incubating for 2 hours at 25 °C. The soluble and insoluble fractions were then obtained by centrifugation. The detergent-soluble fraction was assayed for the presence of MCR-1 by immunoblotting (Fig. 6).

Polymyxin susceptibility testing was performed with the full-length proteins that contain the N-terminal His-tag by determination of MICs using the broth microdilution method according to the CLSI guidelines [24]. The *E. coli* strain BL21(DE3) was used for the susceptibility testing experiments [25].

### Protein expression and purification

Both constructs of the catalytic domain, MCR-1_Δ1–214_ and MCR-1_Δ1–236_ (with the inclusion of an N-terminal His-tag for both constructs), were expressed in *E. coli* BL21DE3 cells; 10 mL of overnight culture was used to inoculate 1 L of LB medium supplemented with 30 μg/mL of kanamycin. The cell culture was then incubated at 37 °C with shaking until it reached an OD_600_ of 0.5–0.7; at this point, protein production was induced by addition of IPTG at a final concentration of 0.5 mM. The culture was then incubated at 25 °C for 20 hours. Afterward, the cells were harvested by centrifugation at 8000 rpm for 40 minutes at 4 °C. The cell pellet was incubated at −20 °C for 4 hours and then resuspended in 20 mL lysis buffer containing 50 mM phosphate pH 7.4, 400 mM NaCl, 40 μM MgCl_2_, and 10 ng/mL DNAse. Cells were ruptured using a French press and the cell lysate was centrifuged at 12,000 rpm for 30 minutes. The supernatant was passed through a 0.22 μm filter and loaded on a HisTrap FF column (GE Healthcare, Pittsburg, PA). The proteins were eluted with a linear gradient of 500 mM imidazole. Protein purity was determined by SDS-PAGE. The protein was concentrated using Vivaspin® Turbo 15 centrifugal filters 30 MWCO (Sartorius, Goettingen, Germany). The N-terminal His-tag was removed by overnight digestion at 4 °C with His-tagged tobacco etch virus protease at a molar ratio of 1:50 (protease:protein). The cleaved sample was again loaded onto a HisTrap FF column for tobacco etch virus separation. The concentration of the protein and buffer exchange to 50 mM HEPES pH 7.2 and 50 mM NaCl was performed with Vivaspin® Turbo 15 centrifugal filters 30 MWCO (Sartorius). It should be noted that only one of the proteins (MCR-1_Δ1–214_) was stable and gave a sufficient amount of pure protein for further crystallography studies. Protein concentration was determined by absorbance measurements at 280 nm using an extinction coefficient of 37,735 M^−1^ cm^−1^ [26].

### Protein crystallization and data collection

Crystal conditions were screened using the vapor diffusion hanging-drop method with 7–8 mg/mL of protein. Crystallization trials were performed with versions of the protein both with and without the His-tag. Drops were set up in 96-well plates at a 1:1 ratio using several commercially available screens, including PEGs and PACT suites from Qiagen (Velno, Netherlands), and PEG/Ion and Crystal Screen suites from Hampton Research (Aliso Viejo, CA). Initial crystals formed only with the protein that had the His-tag removed and produced snowflake-shaped crystals in two conditions: 0.1 M HEPES pH 7.5 and 15 % PEG 20,000 (PEGs suite #30); and 0.2 M zinc acetate dihydrate, 0.1 M sodium cacodylate trihydrate pH 6.5, and 18 % PEG 8000 (Crystal Screen #45). Next, these two conditions were prepared in-house and used to set 96-well plates with the Additive Screen™ (Hampton research) following the manufacturer’s guidelines. Only the second condition containing zinc acetate produced single diffraction-quality crystals in the presence of 3 % w/v D-sorbitol. Crystals were harvested and cryo-protected using a mixture of paraffin oil and peritone (70:30). Crystals were flash-cooled in liquid nitrogen before shipment to the Advance Light Source synchrotron at Berkeley National Laboratory. A 1.32-Å resolution data set was collected on beamline 8.2.1 of the Berkeley Center for Structural Biology in the context of the Collaborative Crystallography Program.

### Data processing, single anomalous diffraction-phasing, and structure refinement

The crystallography data was processed using the CCP4 suite [27]. The images were processed by iMOSFLM and data was scaled using SCALA with the anomalous pairs separated to enable calculation of anomalous difference maps for the zinc containing crystals [28, 29]. Data were then input into the CRANK2 pipeline component of the CCP4 online programs [30]. In brief, free set was defined using SFtools; followed by heavy atom structure factors (F_A_) estimation by SHELXC; substructure determination with SHELXD; substructure improvement with PEAKMAX and REFMAC5; hand determination with MAPRO; density modification with Parrot; model building using Buccaneer and SHELXE; and, finally, an initial refinement with REFMAC5 [31–38]. This was followed by manual inspection and iterative cycles of model building in COOT and crystallographic refinement (including anisotropic B-factors) using PHENIX [37, 39, 40]. The final structure was validated using the PDB_REDO and MolProbity servers [41, 42]. Alignment and RMSD calculations were performed by the SSM procedure [43]. All structural figures were generated with the UCSF Chimera graphics program and the PyMOL Molecular Graphics System (Schrödinger, LLC, New York, NY) [44].

## Additional file

Additional file 1: Figure S1. Amino acid sequence alignment of phosphoethanolamine transferases MCR-1, EptC (*C. jejuni*), and LptA (*N. meningitidis*). (TIF 10272 kb)
