# Supplementary figures and images for: Structure of the catalytic domain of the colistin resistance enzyme MCR-1
Source: BMC Biol. 2016 Sep 21;14:81. doi: 10.1186/s12915-016-0303-0 (PMC5031297; doi:10.1186/s12915-016-0303-0)

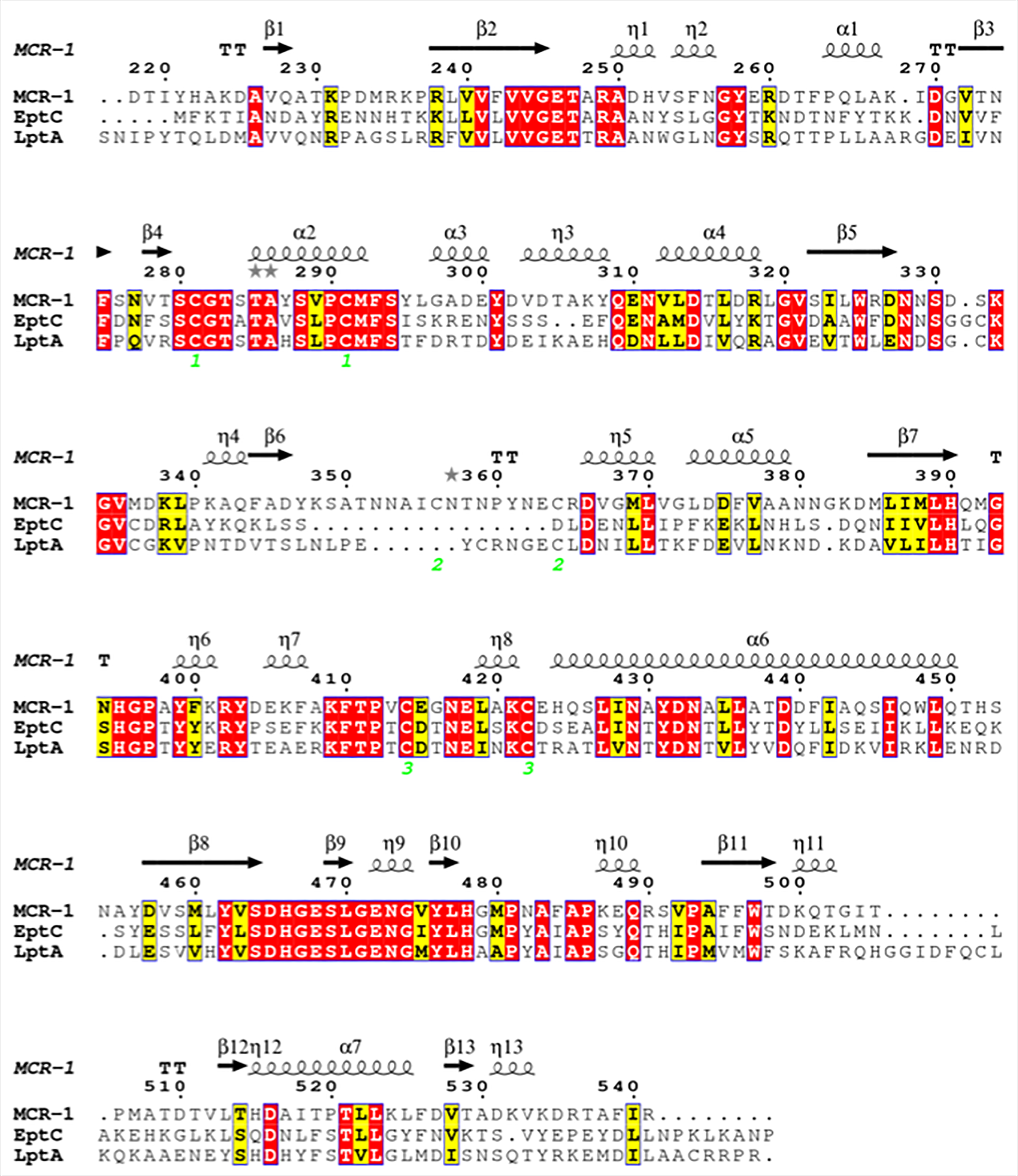

Supplement: Additional file 1: Figure S1. — Amino acid sequence alignment of phosphoethanolamine transferases MCR-1, EptC (C. jejuni), and LptA (N. meningitidis). (TIF 10272 kb) [file 12915_2016_303_MOESM1_ESM.tif]
